# Socio-psychological determinants of public acceptance of technologies: A review

**DOI:** 10.1177/0963662510392485

**Published:** 2012-10

**Authors:** Nidhi Gupta, Arnout R.H. Fischer, Lynn J. Frewer

**Affiliations:** Wageningen University, The Netherlands

**Keywords:** controversy, nanotechnology, public acceptance, socio-psychological determinants, technology

## Abstract

Historically, many technologies have been associated with societal controversies, leading to public rejection of their use. It is therefore important to understand the psychological determinants of societal acceptance of emerging technologies. Socio-psychological determinants of public acceptance of 10 (controversial) technologies are reviewed. The results indicate that there has been an increased interest in and focus on public acceptance of technologies in academia. Risk, trust, perceived benefit, knowledge, individual differences and attitude were found to have been a focus of research in 60% of articles. The results of correspondence analysis suggest that some determinants have been used more extensively in association with some technologies compared to others. As the published research has predominantly been conducted in North America and Europe, research across different cultural contexts internationally is required if globally relevant conclusions are to be reached. Implications for future research are discussed.

## 1. Introduction: Technology and society

Technological advances are continuing to be part of the trajectory of evolving civilization. The quest for knowledge and scientific enquiry has driven humanity to explore developments in science and apply them to human requirements and needs. Technology has been defined as “a technological process, invention or method,” or “the application of knowledge for practical ends” or “the sum of ways in which social groups provide themselves with the material objects of their civilization” (Random House Webster’s College Dictionary, 1997). An example of the last is the definition of technology provided by Mordini (2007) where technology is defined as a “social practice that embodies the capacity of societies to transform themselves by creating and manipulating not only physical objects, but also symbols and cultural forms.” Considerable debate exists over the definition of technology and different approaches to technology (Markus and Robey, 1988; Orlikowski, 1992; Pfaffenberger, 1992; Woolgar, 1991). Social science studies of technology have included perspectives drawn from a large number of disciplines, including, for example, sociology, political science and economics (Klein and Kleinman, 2002; Otway and Winterfeldt, 1982; Pinch and Bijker, 1984; Williams and Edge, 1996). In the research presented here, the focus of the review is confined to social psychological approaches to understanding societal responses to technology.

Evident from the definition by Mordini (2007) is that sequentially evolving technologies are not isolated from the society in which they are embedded, but are integral to the social environment. Increased societal dependency on technologies necessitates the examination of “society–technology” interactions. In this context, it is important to note that on one hand a new technology may bring about radical changes in society, while on the other hand the fate of that technology rests with the society in which it is being applied. A negative societal response may be caused by the fact that, while many technologies deliver benefits to society, they may also introduce new risks (Gunter and Harris, 1998). As a consequence, such developments are often shaped by public controversies and concerns (Horst, 2005).

Public rejection of technologies has frequently resulted in negative consequences for the commercialization of technologies. In particular, unpredicted events and accidents affecting the public have acted as a signal which has resulted in fear and reluctance to adopt certain technologies, and resulted in consumer rejection of the products of these technologies. Perhaps as a consequence, much of the research focused on understanding societal acceptance of technologies has been directed towards risk perception. As a case in point, the Three Mile Island accident sparked controversy and public negativity towards nuclear technology (Chapin and Chapin, 1994; Gilbert, 2007; Van Der Pligt, 1985). Another example is the market introduction of the first generation of genetically modified (GM) food crops, which led to polarized GM food debate internationally (Dale, 2004; Hall, 2007). The intensive societal discussion that followed was detrimental for the adoption and commercialization of GM crops and food products at least in some regions of the world (Aerni, 2005; Batrinou et al., 2005; Frewer, Scholderer and Bredahl, 2003; Klintman, 2002; Trait, 2001). Occurrence of such events and controversies over the use of technology, emphasize the importance of public acceptance in strategic development, application and commercialization of technologies.

Resistance to technologies and factors influencing public acceptance of technologies have generated wide interest in academia, particularly in the arena of social and behavioural research (Sjöberg, 2002). A lot of research has been conducted on risk and (more recently) benefit perceptions and public attitudes as these are believed to be the major factors influencing public acceptance (Alhakami and Slovic, 1994; Barnett, Cooper and Senior, 2007; Costa-Font, Rudisill and Mossialos, 2008; Gaskell et al., 2004; Knight and Warland, 2005; Poortinga and Pidgeon, 2006; Purvis-Roberts, Werner and Frank, 2007; Renn, 2006; Schulte, Hart and Van der Vorst, 2004; Sjöberg and Fromm, 2001; Slovic, 1996; Slovic, Flynn and Layman, 1991). Research in psychology focused on how individuals define risks and understanding the key factors influencing such processes (Ricci et al., 2006), and although originally most emphasis was on cognitive processes (e.g. Kahneman and Tversky, 1979), psychometric study of attitudes towards technological risks and benefits has explored the emotional basis of risk judgements (Fischhoff et al., 1978).

More recently the emotional approach of risk perception has become more dominant with the proposition of a theoretical framework that describes the importance of “affect-heuristic” in guiding risk perceptions and risk-related behaviour (Fischer and De Vries, 2008; Slovic et al., 2002, 2007), and the “risk as feeling” perspective suggests that intuitions experienced at the moment of decision-making can play a vital role in the choice an individual eventually makes (Loewenstein et al., 2001). All of these studies imply that people’s attitudes towards technological risks and benefits are influenced by risk dimensions that have little to do with the possible consequences of the technology. An individual can evaluate a risk cognitively and react to it emotionally. Pesticides, while considered to be the technology driving the “Green Revolution,” and contributing to international improvement in food security, are primarily associated with consumer negativity linked to “negative affect,” or emotional responses, rather than systematic cognitive evaluation of the issues, although these are also a topic of societal discourse (Alhakami and Slovic, 1994). Thus showing that cognitive evaluation and emotional response do not necessarily align. Although these two reactions are interrelated, they have different determinants. Exploring these determinants in detail can facilitate our understanding of the socio-psychological process affecting public acceptance of technology (Pin and Gutteling, 2009).

The aim of this paper is to present an overview of the socio-psychological determinants of relevance to understanding public acceptance of technologies by analyzing the literature in social psychology and risk perception.

The main research question of the study is to identify which socio-psychological determinants of public acceptance of technology have been studied in the social science literature in the field of social psychology and risk perception. To do so the following sub-questions were addressed.

1. What potential socio-psychological determinants which influence public acceptance of technologies have been researched?
2. Are some socio-psychological determinants more relevant to specific technologies?
3. How have the socio-psychological determinants addressed in research of public acceptance of technology developed and changed over time?
4. Are there regional differences in determinants of public acceptance of technologies which have been researched?

## 2. Methods

### The database

A search was conducted using the Scopus (electronic) database in order to identify papers that included information about the determinants of public acceptance of technology. First, a scoping search was conducted to gain information about technologies that have been controversial or have raised discussion about their use. The second, main, search was conducted in order to identify papers focused on these technologies. The time scale for the search was between 1977 and 2008 (one paper of 2009 appeared online as a prepublication) and the last search was done on 12 November 2008. The search was limited to peer-reviewed articles and review papers and the subject area was confined to social science and psychology. Duplicate articles, opinion papers, and articles which did not include relevant data were excluded from the main analysis. A total of 292^1^ research papers were selected for the main analysis which included papers that reanalyzed data using a new analytical approach (*N* = 108). However, these 108 papers were excluded in the regional analysis as country of data collection could not be identified for these papers, therefore leaving 184 papers to be included in the regional analysis. The title, authors, abstract, keywords and bibliographical data of the articles were stored in Endnote. Although Scopus covers over 15,000 journals, a limitation of selecting publications from the Scopus database is that only articles cited in this database, and keywords assigned to the papers by their authors have been included in the review.

### Selection of technologies

The initial scoping exercise was done to quickly scan papers for selecting the technologies in the analysis. Search terms were developed to identify articles that focused on technology and societal controversy. Ten technologies were prominent (although not necessarily evenly distributed in occurrence with times). These were *nuclear technology*, *information and communication technology* (ICT) (including computers and the internet), *mobile phones*, *chemicals used in agriculture* (pesticides and insecticides), *genetic modification*, *genomics*, *cloning*, *hydrogen technology*, *radio frequency identification technology* (RFID) and *nanotechnology*.

After the preliminary scanning, a literature search was conducted to collate papers addressing specific issues with regard to risk perception and its determinants for the selected technologies. The keywords used with each of the technologies were: technologies (as listed above) AND “scare OR fear” AND “controversy” AND “risk perception” AND “consumer acceptance OR consumer response OR consumer acceptability” AND “societal response OR societal acceptance OR societal concern OR social acceptability.” In total 292 papers (Table 1) were found to be relevant, i.e. investigating determinants of social acceptance of technology.

**Table 1.** Distribution of articles and frequency of determinants over technologies.

Technology	No. of articles (out of 292)	Frequency of determinants (out of 558)	Ratio (frequency of determinants/ no. of articles)
Genetic modification	104	210	2.02
Nuclear power	49	99	2.02
ICT	45	93	2.07
Pesticides	30	50	1.67
Nanotechnology	16	30	1.87
Cloning	11	21	1.91
Mobile phones	11	20	1.82
Hydrogen power	7	11	1.57
Genomics	13	14	1.08
RFID	6	10	1.67

### Coding

The year of publication, research question, methodology, and the results were extracted from research articles. The factors influencing public acceptance were recorded from the research articles. These factors were coded into 31 different determinants of technology acceptance using thematic analysis. These were: *Impact (general, positive and negative)*; *Expert versus lay knowledge*; *Affect (general, negative and positive)*; *Impact health (positive and negative)*; *Impact environment (positive and negative)*; *Heuristics*; *Values (general and positive)*; *Perceived risk*; *Perceived benefit*; *Perceived cost*; *Risk management*; *Risk assessment*; *Attitudes (general, positive and negative)*; *Ethics and values*; *Role of societal actors*; *Trust and culpability*; *Concern*; *Citizen knowledge*; *Individual differences*; *Communication*; *Costs*; and *Technology characteristics*.

Countries where data were collected were also coded for all the articles. In total, 39 countries were identified (including research that compared data from consumers in different countries or cultural contexts). These countries were then categorized into seven regions: North-West Europe (UK, Germany, The Netherlands, Switzerland, Belgium, Sweden, Ireland, Norway, Austria, Finland, France, Poland and Denmark); Southern Europe (Romania, Turkey, Italy, Portugal and Spain); North America (USA and Canada); Latin America (Trinidad, Mexico and Argentina); Asia (Singapore, Korea, Hong Kong, Vietnam, Nepal, Bangladesh, Philippines, India, China, Kazakhstan, Malaysia, Japan and Taiwan); Oceania (Australia and New Zealand); and Africa.

### Data analysis

The content of the papers was analyzed on distribution of coded scores across year and region. In addition, correspondence analysis was used to investigate the relationship and trends across different determinants and technologies, to depict the results in categories on a few dimensions (Gurabardhi, Gutteling and Kuttschreuter, 2004; Hoffman and Franke, 1986). To avoid determinants with very low frequencies distorting the analysis, the determinants that appeared only once were merged into super-ordinate categories (the role of societal actors positive and negative were merged into role of societal actors; risk management complete and incomplete were merged into risk management; and risk assessment complete and incomplete were merged into risk assessment).

## 3. Results

### Determinants influencing public acceptance of technologies

Thirty-one potential determinants which emerged from the coding scheme were found to influence public acceptance of new technologies. More than one determinant was found to influence public acceptance in most of the articles. In terms of the technology that was the focus of the research, the most frequently investigated technology was Genetic Modification (*N* = 104). On average an article includes between 1 and 2 determinants (Table 1).

Of the 31 determinants, 6 determinants accounted for about 60% of all determinants mentioned across the sample. Of these, *perceived risk* was found to be the most frequently investigated determinant, and was reported 86 times. *Trust* was used 63 times; *perceived benefit* 51 times; *knowledge* 50 times; *individual differences* 44 times; and *attitudes* 42 times. Other influential determinants were *negative affect* coded 27 times; *technology characteristics* and *role of societal actors* each coded 22 times. In the sample determinants like *negative impact general*, *positive impact general* and *positive attitude* were coded 12 times each and *ethics* and *cost* were coded 11 times. *Communication*, *negative health impact*, *negative environment impact* and *values* were found to be coded about 10 times. Less researched determinants were *expert versus lay knowledge*, *heuristics*, *perceived cost*, *risk management*, *negative attitude*, *general affect*, *concern* and *positive affect* (coded about 6 times each). Determinants that were coded the least number of times (1–2 times) were *positive environment impact*, *risk assessment*, *general impact*, *positive health impact* and *positive value*.

**Figure 1.**
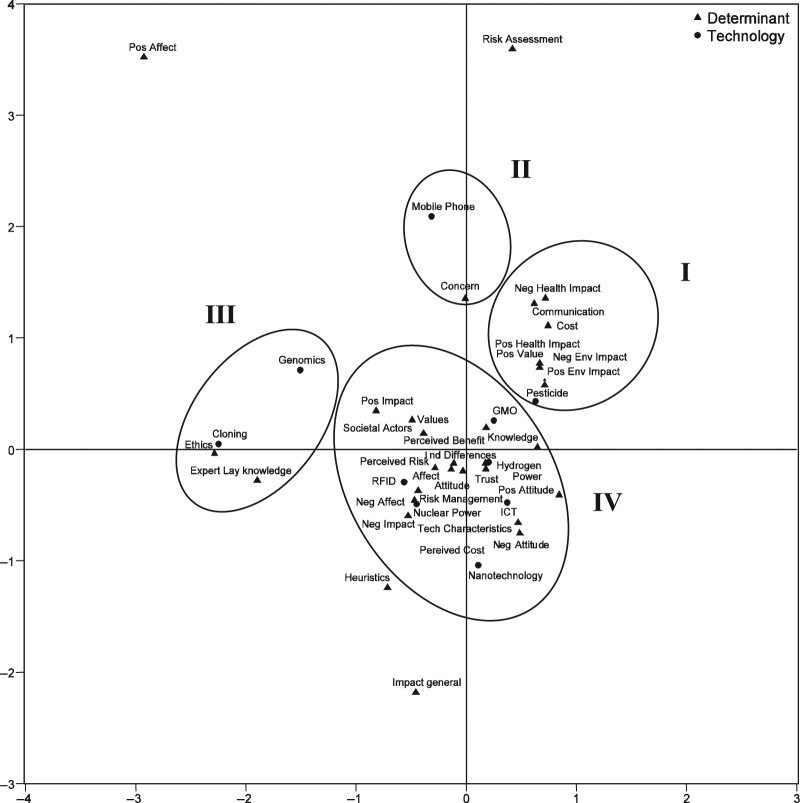
Results of the correspondence analysis of categorized determinants and technologies.

Correspondence analysis between technologies and determinants showed that certain determinants were associated more with specific technologies (χ^2^ = 332.64, *p* = .006; Figure 1). To classify these groups, hierarchical cluster analysis was applied to determine which technologies and determinants are associated more closely with each other. The four clusters identified in the cluster analysis comprised the technologies and the associated determinants. Clusters one and two came out as very clear clusters each including one technology, and one or more determinant. Cluster one showed the association of pesticides with the seven determinants *positive impact (health and environment)*, *negative impact (health and environment)*, *positive value*, *communication* and *cost*. The second cluster suggested that *concern* is associated with mobile phones. In cluster three, genomics and cloning appeared together with two determinants: *ethics* and *expert versus lay knowledge*. While these two determinants were associated strongly with cloning, they were weakly associated with genomics. In contrast to the first three clusters where a clear picture emerges for one single or two related technologies, the fourth cluster consisted of 6 technologies and 17 associated determinants. In this cluster nuclear technology and RFID were closely associated with *values*, *role of societal actors*, *impact general (positive and negative)*, *risk management*, *perceived risk*, *attitude general*, *perceived cost* and *affect (general and negative)*. In the same cluster ICT, nanotechnology, hydrogen power and genetic modification exhibited close association with *attitude (positive and negative)*, *technology characteristics*, *individual differences*, *trust*, *perceived benefits* and *knowledge*. While most of the determinants were found in the four clusters, some determinants were not found to have strong association with any of the technologies. These were: *heuristics*, *impact general*, *risk assessment* and *positive affect*. *Heuristics* and *impact* *general* were related to each other but they did not associate strongly with any of the technologies specifically.

### Temporal trends in research on public acceptance of the technologies

**Figure 2.**
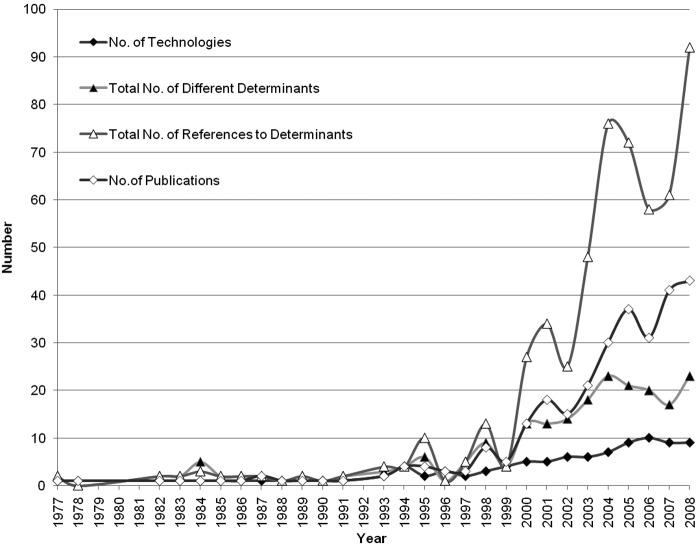
Trends over time (from 1977 to 2008) in number of publications (*N* = 292), technologies studied (*N* = 10), different determinants investigated (*N* = 31) and reference to determinants (*N* = 558) in the sample.

An increase in the number of studies and determinants dealing with public acceptance of technologies occurred over time (Figure 2). A linear regression confirms an increase in publication over the years (*F* (1, 26) = 52.22, *p* < .01, *R*^2^ = .66). Earlier publications focused on nuclear technology (first paper in 1977) and pesticides (first paper in 1988). In 1994 publications on genetic modification started appearing and the topic continues to attract scholarly attention, making it the most extensively researched upon technology. Research articles on hydrogen power, cloning, genomics and RFID were sporadic. Most recent in these technologies is nanotechnology, with papers being published in 2006, 2007 and 2008.

**Figure 3.**
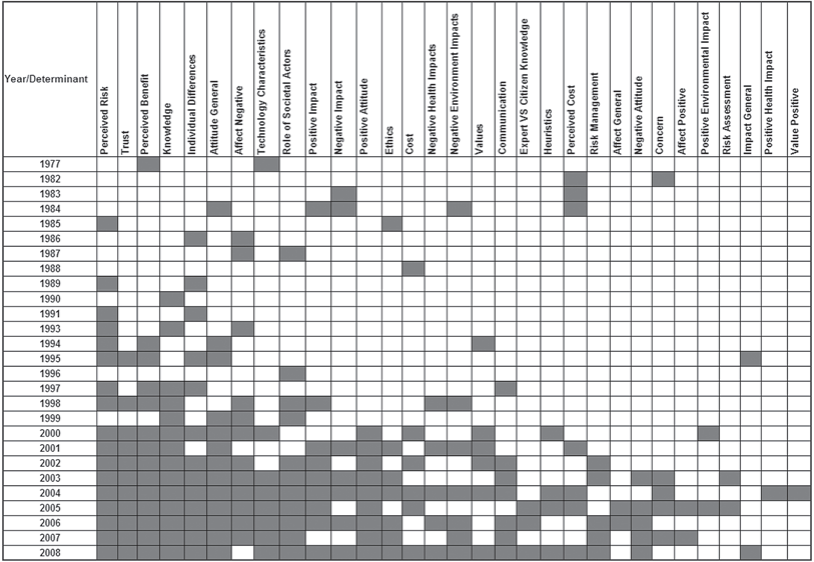
Coverage of determinants over the years (each grey box refers to multiple occurrences of determinants in each year).

Over time, the number of determinants that have been investigated has increased (Figure 3), implying that research directed towards understanding public acceptance of technologies is becoming increasingly sophisticated. From Figure 3 we can see that the models used to predict public acceptance are getting more complex, with a wide coverage of determinants influencing technology acceptance. “Classical” determinants, for example *risk perception*, *benefit perception*, *trust*, *knowledge*, *attitude*, *negative impact* and *individual differences* continue to be included in research designs. In addition new determinants (such as *heuristics*, *concern*, *risk assessment*, *positive impact* and *positive value*) have been the topic of more recent research. In terms of risk and benefit perception, *perceived risk* was cited more often than *perceived benefit*, showing researcher prioritization of risk perception over and above benefit perception as an important determinant of consumer acceptance.

### Regional trends in research on public acceptance

Regional trends in research on various determinants were examined for 184 research articles that included information that enabled the identification of country of collection (Table 2). Looking at the frequency of determinants investigated in different regions across the world, research originating in North-West Europe investigated the greatest number of determinants (44%). This was followed by research originating in North America (30%). Fewer determinants were investigated in research studies originating in Asia and Southern Europe, the least were reported for research originating in Latin America and Oceania. This sharp decline in frequency of determinants is, in part, attributable to fewer publications addressing few technologies in these regions. Of the 184 research articles, 19 articles were comparative, as data were collected in different countries or regions. However, these articles were again dominated by data originating in North-West Europe and North America.

**Table 2.** Regional distribution of articles and determinants on public acceptance.

Region	No. of technologies covered	No. of determinants
North-West Europe	10	29
North America	9	27
Asia	5	16
Southern Europe	5	18
Latin America	1	8
Africa	2	8
Oceania	3	9

## 4. Discussion and conclusion

Public acceptance of technologies continues to be a focus of scholarly attention, as demonstrated by the steady rise in the number of publications and determinants investigated that are found to impact the acceptance. Regional trends show that most of the research has been carried out in North America and North-West Europe. While this may be in part, because the search was limited to the English language, it is nevertheless clear from this that most of this type of research is concentrated in the developed world. More research is needed in developing countries and with developing economies, to present a more comprehensive picture of societal response to new technologies.

Of the ten controversial technologies studied, research was most frequently focused on nuclear power, genetic modification and ICT compared to genomics, cloning, mobile phones and hydrogen power. The study of public acceptance of nanotechnology and RFID has only recently been initiated, in line with the recency of technological advances, and it is therefore too early to judge whether research into these technologies will provide major contributions to the technology acceptance literature. The publication trend for different technologies can be partly related to the year of their introduction or commercialization. Discussion about the consequences of using nuclear technology has been a topic of public debate since World War 2. Application of synthetic pesticides in agriculture drew public criticism in 1962 with the publication of *Silent Spring* by Rachel Carson, inspiring widespread public concerns associated with pesticide use and environmental pollution (Kroll, 2001; Pollock, 2001). The consequences of using genetic modification escalated the already existing public debate on the use of new technologies in 1994 with commercialization of genetically modified food crops and products. Ever since its introduction, the technology has been exposed to media attention and societal debate about its merits or otherwise (Bauer, 2002, 2007). Research focused on the application of cloning technology started appearing around 1997 when the first cloned higher animal “Dolly” (a sheep) was developed (Bauer, 2002). An important point is that research into public acceptance of new technologies has tended to occur post-commercialization, when public concerns have begun to arise. In the case of nanotechnology, the discussion has been initiated at about the same time, in response to concerns from developers about public negativity to its application. This indicates a shift in focus on public acceptance of technologies, from *post hoc* studies to a more proactive effort to identify public opinions and values prior to commercialization. The extent to which such information will be used to shape science strategy and the specific application of nanotechnology remains to be evaluated.

Social science analysis focused on public acceptance of specific technologies is typically conducted after the technology has been introduced and commercialized, and subsequently has been associated with societal disquiet or negativity. Thus, in the past, it would appear social science research funding has been allocated to those technologies that have become societally controversial. In order to better understand the process of technology acceptance in society, research into non-controversial technologies might be applied to identify what factors drive societal acceptance (assuming that comparative analysis can be applied across technological areas that are inherently associated with different levels of impact or political controversy). It would also resolve whether the inherently “dramatic” qualities of some technologies also drive researcher interest, which in turn drives funding cycles and societal discourse about the technologies in question.

It is recognized that the socio-political context in which technologies are embedded also shapes public debate and acceptance of these same technologies. Further discussion of this is beyond the scope of the current research paper, as defined by the original research question. The question of why some technologies become societally controversial, whereas others do not, is worthy of further research.

The sophistication of the socio-psychological factors used to assess attitudes has also increased with time, reflecting theoretical advances in this area. *Perceived risk*, *perceived benefit*, *trust and culpability*, *knowledge*, *individual differences* and *attitude* are traditionally the most often reported or cited determinants, and these remain dominant. Temporal analysis of the data indicates that the postulated models explaining public acceptance have increased in complexity, by adding, rather than replacing determinants. Determinants that were found to influence public acceptance of one technology contribute in shaping the acceptance for other technologies. The analysis has demonstrated that the number of social psychological determinants investigated in the context of technology acceptance has increased, perhaps reflecting theoretical advances in understanding public responses to technologies. Some determinants (for example, *positive affect*, *risk assessment* and *heuristics*) have been less frequently studied. A systematic critique of the relative predictive capacity of these different determinants is not currently available. A first step in the development of such a systematic review would be the simultaneous analysis of all potential determinants in a single study, or (possibly) through application of formal meta-analysis if appropriate data are available. This is a topic worthy of future investigation.

The temporal analysis has also confirmed that research that has focused on the individual as a “non-rational” actor has increased. This research suggests considerable support for the socio-psychological determinants of acceptance of technology underpinning lay opinion, as well as providing an explanation as to why these might differ from expert views. Further investigation into the disparity between lay and expert opinions of technology may systematically contrast the extent to which the different determinants predict technology acceptance in each group.

Certain determinants are seen to have more impact on public acceptance of specific technologies. Pesticides were mainly associated with health and the environment, cloning and genomics with ethics; while a large group of technologies was associated with most of the remaining determinants. This association between certain types of technologies and determinants to some extent can help us to understand and predict the factors that will set the stage for discussion of new and emerging technologies.

In this paper a specific class of technologies has been investigated, that is, those technologies that have been enabling a myriad of applications with the potential to change society, as the impact of these applications ripples through society. These technologies can be called “transformative,” as they have the power to transform society by introducing completely new social phenomena. Previous transformative technologies (agricultural technology, printing, aircraft and vaccinations) have had lasting effects on human values, power structures and ideas and acted as potential drivers of socio-economic, political and institutional change (Crow and Sarewitz, 2001; Dolata, 2009; Shneiderman, 1998). The emergence of the technologies reviewed in this paper not only fuelled the engines of economy and growth, but also raised critical issues of political and military influence (e.g. nuclear power), international competition (e.g. GMO), environmental crisis (e.g. pesticides), ethical debates in relation to the discussions on the ethical acceptability of human control over and manipulation of nature (cloning and genomics), social changes resulting from expectations of being connected 24 hours a day, seven days a week (mobile phones) and protection of privacy (RFID, ICT).

Nanotechnology has the potential to become a transformative technology. It is among the recent emerging technologies which have been the focus of attention on the part of stakeholders, opinion leaders and media discussion. On one hand it presents unmatched opportunities to develop new products and services, and may result in longevity, public health benefits, and more sustainable production, but on the other raises concern, fear and anxiety among the public (Romig et al., 2007; Schütz and Wiedemann, 2008; Siegrist et al., 2007). Understanding the socio-psychological factors would allow contextualization of its development and implementation, and potentially facilitate allocation of resources in areas of application relevant to the wider needs of society (Renn and Roco, 2006; Roco, 2003).

Future research needs to explore the interrelationships between determinants, particularly those which have emerged as being influential in recent years, such as the relationship between perceived risk and benefit, but also identify the knowledge gaps and explore other psychological factors that have recently started appearing in the literature such as heuristics and affective responses.

## Notes

1. Upon email request to the authors, a list of all 292 articles is available.
